# Development and psychometric evaluation of the female infertility stigma instrument (ISI-F): protocol for a mixed method study

**DOI:** 10.1186/s12978-020-0904-5

**Published:** 2020-05-24

**Authors:** Mahboubeh Taebi, Nourossadat Kariman, Ali Montazeri, Hamid Alavi Majd

**Affiliations:** 1 Student Research Committee, Department of Midwifery and Reproductive Health, School of Nursing and Midwifery, Shahid Beheshti University of Medical Sciences, Tehran, Iran; 2 School of Nursing and Midwifery, Isfahan University of Medical Sciences, Isfahan, Iran; 3 Midwifery and Reproductive Health Research Center, Department of Midwifery and Reproductive Health, School of Nursing and Midwifery, Shahid Beheshti University of Medical Sciences, Tehran, Iran; 4 Health Metrics Research Centre, Iranian Institute for Health Sciences Research, ACECR, Tehran, Iran; 5 Department of Biostatistics, School of Allied Medical Sciences, Shahid Beheshti University of Medical Sciences, Tehran, Iran

**Keywords:** Study protocol, Sequential exploratory mixed-method study, Validity, Reliability, Psychometric evaluation, Infertility, Female Infertility Stigma Instrument, ISI-F

## Abstract

**Background:**

Infertility stigma is one of the greatest challenges in most societies for reproduction and sexual health of infertile women. Since no specific tool exists for assessing the infertility stigma in women, this study would be conducted to develop and evaluate the psychometric properties of Female Infertility Stigma Instrument (ISI-F).

**Methods:**

This is a mixed method study with sequential exploratory design (qualitative and quantitative phase). In the first qualitative phase, semi-structured interviews would be performed with infertile female who had experienced infertility whithout any psychological disorder. Women who are eligible for participating in the study will be selected using purposeful sampling method with maximum variation in terms of age, education, occupation and infertility duration. Data would be analyzed using conventional content analysis and in this phase the primary item pool will be developed for the Female Infertility Stigma Instrument (ISI-F). In the quantitative phase, the psychometric properties of the Instrument would be evaluated, including the content, face and construct validity as well as reliability via the internal consistency and stability. The psychometric properties described in the COSMIN checklist will be utilized for designing the instrument.

**Discussion:**

Developing a valid and reliable scale for Female Infertility Stigma Instrument (ISI-F) would be helpful for future studies to assess the status of this situation. It also helps planning interventional studies for improvement of the reproductive health of infertile women.

## Plain English summary

Infertility is a phenomena which is associated with various psychological and social tensions for women. For many of the infertile women, infertility is a hidden label or stigma which is associated with a sense of shame and secrecy. Therefore, the stigma would make the infertile person unable to accept themselves like others due to their social experiences. The label of infertility would make the infertile women have negative perception of themselves and become socially isolated. Considering the adverse effects of infertility on the mental and psychological status and relationship of people, existence of an appropriate tool that could evaluate the current status and help related researches in the future seems necessary. Considering lack of a standard Instrument for evaluating the stigma of infertility in women, the present consecutive mixed study with exploratory approach would be conducted for designing and psychometric evaluation of the tool for evaluation of the infertility stigma in infertile women. This study would be conducted in two consecutive phases. In the first stage, items of the Instrument would be achieved by interviewing women with infertility who has no mental and psychological problems. Then, they would be used for structuring and designing a questionnaire. In the second stage, validity and reliability of the primary Instrument would be confirmed by infertile women (300 women). Consequently, a valid and reliable tool would be extracted for evaluating the stigma of infertility in women.

## Background

Infertility is a common global problem and is considered as one of the greatest challenges of the reproductive ages. Infertility is inability to get pregnant after 12 months of having regular sexual intercourse without any kind of protection [1, 2]. Although it is complicated to estimate the rate of infertility due to existence of female and male factors [3], the prevalence of infertility has no significant difference between various ethnic and racial groups [2]. In general, 8 to 12% of the couples of the reproductive ages around the world are suffering from infertility [4]. According to the reports by WHO, more than 10% of women are affected by infertility [3].

In most of the societies, getting prepared for having a child after marriage is common and even in developed countries, having a child of your own is considered a significant achievement [5, 6]. Therefore, infertility could be considered as a crisis in the couple’s life [7] that could cause disruption in the family’s stability [8, 9]. Due to the real or unreal imagination of feeling unaccepted by the society or lack of sympathy by the family and friends, the infertile couple mostly feel separated from the world of the fertile couples [10]. Feeling of isolation, social stigma, losing control and being flawed along with infertility would become the central part of the identity of infertile couples [11, 12].

For most women, infertility is a hidden stigma which is associated with the feeling of shame and secrecy [13]. Stigma is defined as a negative feeling of being different in the society compared to others and being against the social norms. If infertility would be experienced as a stigma, it would deprive the infertile person from the supports that they could receive and would cause depression, anxiety and stress [14, 15], feeling of guilt [16] and disorder in relationships [17]. It could also cause disturbance, decreased self-esteem and self-efficacy and tendency toward internal stigma [13, 18].

Since for defining their identity and meaning of life, usually women would consider a space for becoming mother and they are mostly prepared to sacrifice their opportunities for raising their child, women would consider themselves more vulnerable than men against infertility and infertile women would experience more stigma than infertile men [5, 6] Infertile women would experience more mental pressure than infertile men and would be labeled repeatedly for being infertile and not having a child [19, 20]. However, if a man, for any reason, could not become a father, the would have other sources for satisfying their sense of achievement and could compensate for lack of success in fertility by their social and occupational activities [21].

Some studies have been performed for evaluating the stigma of infertility [14, 16, 20, 22–24] using general tools for evaluation of stigma and by adding the term “infertility” to these general tools, the stigma of infertility has been evaluated. So designing and developing a tool that would specifically evaluate the stigma of infertility among infertile women seems necessary. The present mixed method study with qualitative content analysis approach could deeply evaluate the perceived stigma by infertile women. The present study would be conducted for designing and psychometric evaluation of the Female Infertility Stigma Instrument (ISI-F).

### Objectives

The objectives of each phase are as following.

### Objectives of the first phase: qualitative study

- Exploring the concept of infertility stigma in infertile women.

- Developing a comprehensive item pool for ISI-F.

### Objectives of the second phase: quantitative study

- Evaluation of the content validity (qualitative and quantitative) of ISI-F.

- Evaluation of the face Validity (qualitative and quantitative) of ISI-F.

- Evaluation of the construct validity of ISI-F Using exploratory facor analysis.

- Evaluation of the reliability of ISI-F using internal consistency and stability assessment methods.

## Methods/design

**Fig. 1:**
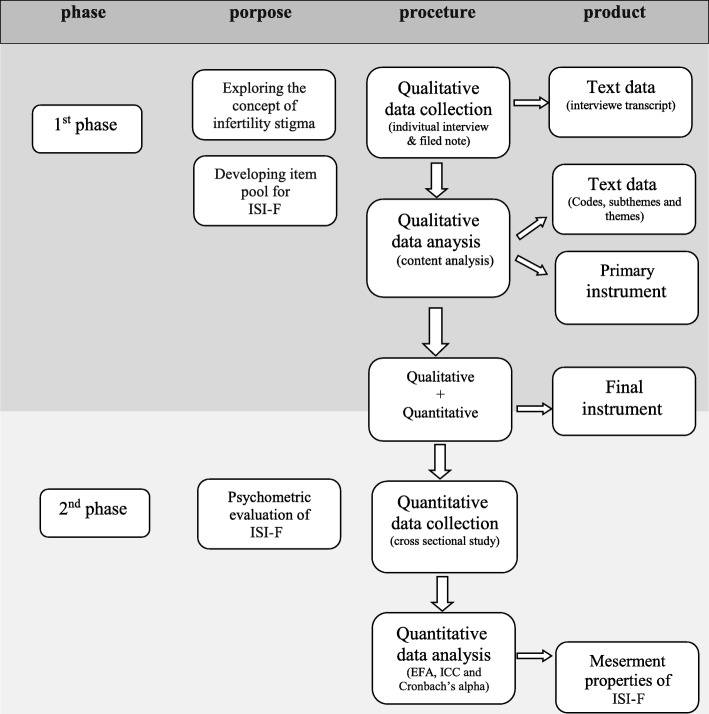
The data collection process illustrating the sequential exploratory mixed method study. ISI-F; Female Infertility Stigma Instrument

This is a sequential exploratory mixed-method study, with the qualitative-quantitative sequencing design (Fig. 1). In the qualitative phase, the concept of infertility stigma will be explored based on infertile female experiences and literature review and then, the primary items of ISI-F would be developed. In the second phase (the quantitative phase), the psychometric properties of the instrument would be assessed.

### The qualitative phase

This phase of the present study will be carried out using a qualitative content analysis method. This study is designed to answer the following question “What is the concept of infertility stigma in infertile women?”

### Data collection

Data will be collected through in-depth semi-structured interviews with infertile women and taking field notes. Women who are eligible for participating in the study will be selected using purposeful sampling method with maximum variation in terms of age, education, occupation and infertility duration. While collecting the data, the interviews will be analyzed using a conventional qualitative content analysis method [25]. In qualitative studies, sample size is unpredictable and sampling will be continued until data saturation occurs [26].

### Characteristic of the participants

Study population consists of women with known primary female infertility who had experienced infertility whithout any psychological disorder. Women who refer to Isfahan fertility and infertility centre, Isfahan, Iran, will be enroled in the study voluntarily and with informed consents.

### Research setting

The interviews will be conducted individually at selected time and location by the participants; also a private room in Isfahan fertility and infertility centre will be considered for the interviews due to its accessiblity, comfort and ease of use for the participant.

### Data analysis

Content analysis with conventional approach will be utilized along with data collection through the Graneheim and Lundman approach (2004) [27]. Transcription, analysis and coding of each interview will be performed before the beginning of the next interview. Codes, sub- categories, categories and themes will be derived from the transcripted data. The combinations of related initial codes will be labeled to form sub-categories and categories. Finally, the latent meaning of the text and the main themes will be extracted by consensus between researchers, until the concept of stigma in infertile women will be obtained. The extracted themes and main categories, besides the existing literature and instruments, will be used to generate the primary item pool for ISI-F.

### The quantitative phase

This phase of the study will evaluate the psychometric criteria of ISI-F including content, face and construct validity, as well as reliability (internal consistency and stability).

### Content validity

Content validation plays a primary role in the development of any new instrument. The qualitative and quantitative methods will be used to determine the content validity of ISI-F.

In the qualitative content validity method, the opinions of ten experts in the field of qualitative research, instrument development, Psychology, midwifery and reproductive health will be used to assess the proper grammar, appropriate and correct words and items’ scoring. Quantitative content validity will be evaluated by the content validity ratio (CVR) and content validity index (CVI) [28].

For CVR calculation, experts will be invited to assess item essentiality. The score of each item would be considered within a three-degree range of “not essential, useful but not essential, essential” from 1 to 3 points. CVR varies between 1 and − 1. Higher scores indicate further agreement of the experts on the essentiality of an item in a tool. The formula is:

CVR = (Ne - N/2)/ (N/2).

Ne = the number of experts indicating “essential”.

N = the total number of experts.

The total score of CVR is determined by Lawshe Table (1975) and based on the number of the expert [29]. In this study 10 experts will be attended, so any item with a CVR of more than 0.62 will be accepted.

CVI is the most widely reported index for quantitative content validity in tool development [30]. CVI can be computed using the Item-CVI (I-CVI) and the Scale level-CVI (S-CVI). Experts are asked to rate the relevancy of each item on a 4-point scale from 1 to 4 respectively (not relevant, somewhat relevant, quite relevant, highly relevant). I-CVI will be computed by dividing the number of experts giving a rating score of either 3 or 4 by the total number of experts. Values of CVI more than 0.79 would show the item is relevant [31]. Average of the I-CVIs for all items on the scale will be assessed by S-CVI via mean scores for content validity index. S-CVI values of greater than 0.9 indicating that have excellent content validity [32].

### Face validity

The face validity of this study will be assessed by quantitative and qualitative method.

In the qualitative approach, 10 face-to-face interviews would be conducted with the target group and the difficulty level, proportion and ambiguity of the items would be examined. After correction, the quantitative approach will be perfromed. Quantitative face validity assessment will be done via the item impact measurement technique. 10 infertile women will score the importance of each item with a 5-point Likert scale, from slightly important (score1) to very important (score 5). The item impact score is calculated by the following formula:

Impact Score = Frequency(%) × Importance.

Importance = Patients who will check the options 4 and 5.

The impact score of more than 1.5 will show that the item is acceptable and will be chosen for further analysis [33].

### Construct validity

Exploratory factor analysis (EFA) will be used to evaluate the construct validity and extract the latent constructs of ISI-F [34].

### Sampling and sample size

Study population will consist of the women who referred to Isfahan Fertility and Infertility Center with known primary female infertility, had experienced infertility whithout any psychological disorder.

The sample sieze would be based on the number of items extracted at the first phase of the study. The number of samples is relevent to the number of items and the proportion of N/K should not be less than 5/1 [26]. Therefore, the number of samples would be calculated based on the extracted items and to the maximum. At this phase, sampling would be conducted using convinient sampling method.

### Statistical data analysis

In order to evaluate the adequacy of sampling to perform exploratory factor analysis, sample size is important, so KMO test and Bartlett’s sphericity test will be used to confirm the adequacy of sampling in EFA. The KMO index ranges from 0 to 1. KMO more than 0.7 is interpreted as acceptable and large sample size that is suitable for EFA [28, 34]. The Bartlett’s Test of Sphericity should have significant results (*p* < 0.05). To determine the best structure, the eigenvalue greater than one and with factor loading equal to or greater than 0.4 will be applied [35].

Moreover, statistical analyses will be performed by running exploratory factor analysis, Pearson correlation analysis, Cronbach’s alpha model, intraclass correlation coefficient and standard error measurement [34]. All the statistical calculations would be performed using SPSS software and for all the tests a maximum error of 5% will be accepted.

### Reliability

Internal consistency and stability will be used to verify the reliability of ISI-F.

Internal consistency would be estimated by computing Cronbach’s alpha coefficient for ISI-F and its subscales. The alpha values of 0.70 or above would be considered acceptable.

Test-retest reliability of ISI-F and its subscale for two-week interval will be estimated by intraclass correlation coefficient (ICC). ICC values of 0.7–0.8 will be considered as having suitable stability [28, 34]. Items that do not have good reliability will not be included in factor analysis to check the construct validity. The psychometric properties described in the COSMIN (consensus-based standards for the selection of the health status measurement instrument) [36] checklist will be utilized for designing the instrument.

## Discussion

Infertility could have harmful social and mental outcomes for the women; from rejection and divorce to social stigmas that could cause isolation and mental disturbance [14, 37]. Stigma, which is defined as crushed identity and being inappropriate, is associated with the social and mental aspects of infertility and, based on their social experiences, it would cause the infertile individuals unable to accept themselves like others, have negative perception of themselves and be socially isolated [6, 38]. Considering the adverse effect of infertility on the mental and psychological status ad relationship of people [10, 17, 39, 40], it requires specific tools that, along with having sufficient validity and reliability, would be able to efficiently evaluate the perceived stigma of infertility for understanding the current situation. It should also help planning and taking appropriate counseling policies for improving the fertility health in infertile women. It is possible that in the present study, some of the women would not express all of their perceived feelings related to infertility which would be one of the limitation of this study. However, the efforts would be toward gaining the trust of the participants and establishing a relationship with them for resolving this limitation. Some of the strong points of the present study is using sequential exploratory mixed method design and selecting a large number of participants from infertile women with various duration of infertility and various social status.

## Data Availability

Not applicable.

## Abbreviations

ISI-F: Female Infertility Stigma Instrument
CVR: Content Validity Ratio
CVI: Content Validity Index
EFA: Exploratory Factor Analysis
ICC: Intraclass Correlation Coefficient
SPSS: Statistical Package for Social Science
COSMIN: COnsensus-based Standards for the selection of health Measurement Instruments
